# The Absence of CD47 Promotes Nerve Fiber Growth from Cultured Ventral Mesencephalic Dopamine Neurons

**DOI:** 10.1371/journal.pone.0045218

**Published:** 2012-09-26

**Authors:** Franziska Marschinke, Sanaz Hashemian, Takashi Matozaki, Per-Arne Oldenborg, Ingrid Strömberg

**Affiliations:** 1 Department of Integrative Medical Biology, Umeå University, Umeå, Sweden; 2 Division of Molecular and Cellular Signaling, Department of Biochemistry and Molecular Biology, Kobe University Graduate School of Medicine, Kobe, Japan; University of North Dakota, United States of America

## Abstract

In ventral mesencephalic organotypic tissue cultures, two timely separated sequences of nerve fiber growth have been observed. The first appearing nerve fiber pattern is a long-distance outgrowth that occurs before astrocytes start to proliferate and migrate to form an astrocytic monolayer that finally surrounds the tissue slice. These long-distance growing nerve fibers are retracted as the astrocytes migrate, and are followed by a secondary outgrowth. The secondary outgrowth is persistent in time but reaches short distances, comparable with outgrowth seen from a dopaminergic graft implanted to the brain. The present study was focused on the interaction between the astrocytes and the long-distance growing non-glial associated nerve fibers. Cross talk between astroglia and neurite formation might occur through the integrin-associated protein CD47. CD47 serves as a ligand for signal regulatory protein (SIRP) α and as a receptor for the extracellular matrix protein thrombospondin-1 (TSP-1). Embryonic day 14 ventral mesencephalic tissue from CD47^+/+^ and CD47^−/−^ mice was used to investigate astrocytic migration and the tyrosine hydroxylase (TH) –positive outgrowth that occurred remote from the astrocytes. TH-immunohistochemistry demonstrated that the non-glial-associated nerve fiber outgrowth in CD47^−/−^ cultures reached significantly longer distances and higher density compared to nerve fibers formed in CD47^+/+^ cultures at 14 days *in vitro*. These nerve fibers often had a dotted appearance in CD47^+/+^ cultures. No difference in the astrocytic migration was observed. Further investigations revealed that the presence of CD47 in control culture did neither hamper non-glial-associated growth through SIRPα nor through TSP-1 since similar outgrowth was found in SIRPα mutant cultures and in CD47^+/+^ cultures treated with blocking antibodies against the TSP-1, respectively, as in the control cultures. In conclusion, long-distance growing nerve fiber formation is promoted by the absence of CD47, even though the presence of astrocytes is not inhibited.

## Introduction

Regeneration in the adult brain involves neurotrophic factors, neuroinhibitors, cell adhesion, and extracellular matrix molecules that may affect the regenerative process. These molecules are often produced by the glial cells and affects the nerve regeneration for instance in the spinal cord after injury, where regeneration beyond the glial scar is problematic. Another field for regenerative studies is regeneration induced by neural grafts in neurodegenerative disorders. Much effort has been dedicated to study reinnervation of the striatum induced by transplanted dopaminergic cells in animal models of Parkinson's disease [1], [2]. In this situation no strong correlation to inhibited graft outgrowth has been accounted to the astrocytic influence, still graft outgrowth is limited to small zones surrounding the transplants, which limits the effect of the transplant, unless multiple graft sites are made to cover most of the dopamine-denervated portion of the striatum [3]. To investigate what influences nerve fiber growth and what may make the graft outgrowth to halt at a certain distance from the grafted tissue, organotypic tissue cultures have been employed.

In ventral mesencephalic organotypic cultures, two morphologically different types of nerve fiber growth patterns have been observed [4]. The different waves of outgrowth depend on astroglia, i.e. either in the absence of astroglia or in the close association with astrocytes [4], [5]. The non-glial-associated outgrowth appears early, already after 2–3 days *in vitro* (DIV), without the presence of glial cell bodies, and retracts usually after some weeks. The later appearing nerve fiber formation is found in the presence of astroglia and is persistent over time. Based on studies using a mitotic inhibitor, a strong relationship between the two growth patterns has been suggested [6]. Thus, when promoting astrocytic migration, the non-glial-associated outgrowth disappears, while it is present when inhibiting astrocytic migration [6], [7]. Adding neurotrophic factor to the medium enhances the density of glial-associated nerve fibers, while the non-glial-associated growth is not affected [8]. Interestingly, when the presence of non-glial-associated growth is promoted, nerve fibers continuously elongate and reach distances of several mm. The maximal distance that the migrating astroglia and glial-associated nerve fibers reach is around 1 mm, which is approximately the same distance that graft outgrowth reaches in the dopamine-depleted striatum [5], [9], [10]. Thus, the interplay between astrocytes and nerve fiber growth appears important for the distance that the nerve fibers may grow during regeneration.

Extracellular matrix molecules, integrins or integrin-associated protein, also known as CD47, are factors that are expressed by the astrocytes. It is known that the extracellular matrix proteins, such as the proteoglycans, widely affect nerve fiber outgrowth, however, little is known about the effects of CD47 [11], [12], [13], [14]. CD47 is widely expressed in the brain, especially abundant in synapse-rich regions, and its expression increases during postnatal development [15], [16], [17], [18]. CD47 act as a ligand for signal regulatory protein alpha (SIRPα), a neural adhesion molecule, also known as P84, BIT, (brain immunoglobulin-like molecule with tyrosine-based activation motifs), or SHPS-1 (SHP substrate 1), and was first localized to neurons [16], while it was later also detected in immune cells like monocytes, granulocytes and macrophages [19]. The neurotrophic factor brain-derived neurotrophic factor, which is potent for dopamine neurons, exerts its effect through SHP-2 by affecting the phosphorylation of SIRPα [20], [21], [22]. Moreover, CD47 act as a receptor for thrombospondin-1 (TSP-1), belonging to a family of extracellular matrix glycoproteins, which is widely expressed in the developing and adult brain and exert a wide range of effects on cell behavior such as migration, adhesion, and neurite outgrowth [23], [24], [25]. Overexpression of CD47 improves dendritic growth and affects synaptic proteins, while CD47 gene deletion improves regeneration in the spinal cord [26], [27], [28]. Thus, the results gives contradicting information and to further elucidate the role of CD47 in affecting nerve fiber formation, especially the non-glial-associated nerve fiber growth, this study was undertaken to investigate nerve fiber growth in organotypic slice cultures of fetal ventral mesencephalon derived from CD47 gene deleted mice and to study the effects of TSP-1 and SIRPα.

## Materials and Methods

### Animals and Animal Keeping

The generation of CD47^−/−^ Balb/c mice has previously been described [29]. The mice were backcrossed to Balb/c (Jackson Laboratory, Bar Harbor, ME) for 16 generations, and their homozygous littermates were used from our own breeding colony. In addition, SIRPα-mutant C57BL/6 mice, lacking most of the SIRPα cytoplasmic domain [30], were backcrossed to C57BL/6 for 10 generations, and their homozygous littermates were used from our own breeding colony. Mice were kept in a 12/12 h light/dark cycle with access to food and water *ad libitum*. Mating was made over one night, and fetuses collected at embryonic day 14. These experiments had been approved by the local ethics committee, Umeå Ethics Committee for Animal Studies (permit number: A23-08).

**Figure 1:**
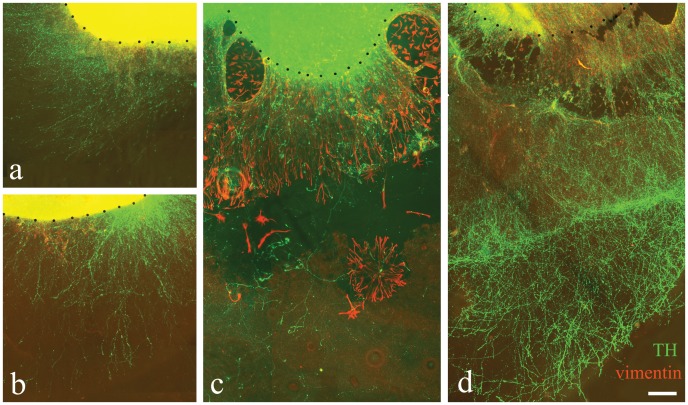
TH-positive nerve fiber outgrowth and astrocytic migration in CD47^−/−^ cultures. TH-positive nerve fiber outgrowth and vimentin–positive astrocytic migration from CD47^+/+^ (a, c) and CD47^−/−^ (b, d) VM cultures after 7 (a, b) and 14 (c, d) DIV. The astrocytic migration was shorter at 7 DIV as compared to 14 DIV. Short TH-positive nerve fiber outgrowth was observed in CD47^+/+^ (a) and CD47^−/−^ (b) cultures at 7 DIV, while at 14 DIV the TH-positive nerve fibers had reached over longer distances and with higher density in CD47^−/−^ cultures (d) compared to control cultures (c). The dotted line delineates the border of the tissue slice. Scale bar = 100 µm.

**Figure 2:**
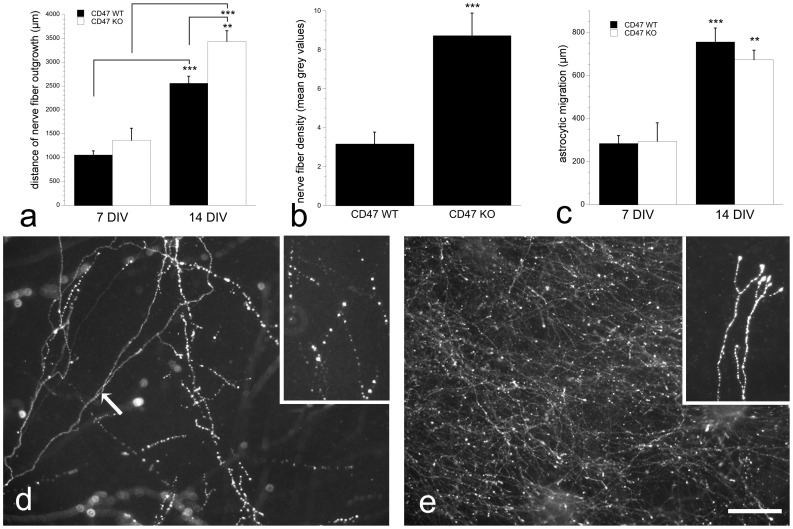
Differences in nerve fiber outgrowth in CD^−/−^ compared to CD^+/+^ cultures. The distance that TH-positive nerve fibers reached was significantly enhanced over time in both CD47^+/+^ and CD47^−/−^ cultures, while nerve fiber outgrowth was significantly longer in CD47^−/−^ cultures at 14 DIV (a). Measurements of nerve fiber density at 14 DIV showed significantly increased density of TH-positive nerves in CD47^−/−^ cultures (b). The length that the astrocytes migrated from the tissue slice was similar in cultures from both genotypes, but affected by the time such that the astrocytes reach longer distances at 14 DIV compared to 7 DIV (c). TH-immunohistochemistry demonstrating non-glial-associated nerve fiber growth in CD47^+/+^ (d) and CD47^−/−^ (e) cultures at 14 DIV. Nerve fibers often have a dotted appearance in CD47^+/+^ cultures (d insert) even though smooth, thin fibers also were present (arrow), while in cultures derived from CD47^−/−^ tissue the nerve fibers were thin with varicosities and enlarged nerve endings (e insert) were frequently present. **p<0.01, *** p<0.001, significances in (c) refers to changes within the same genotype. Scale bar = d, e = 50 µm, inserts in d and e = 25 µm.

**Figure 3:**
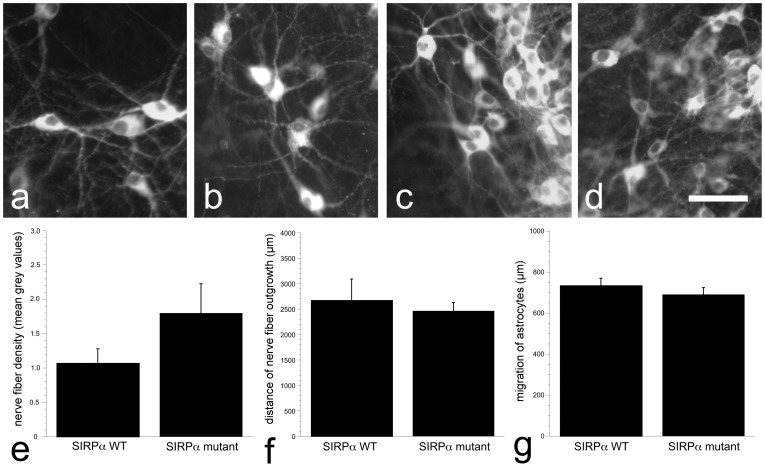
Survival of TH-positive neurons and outgrowth from SIRPα mutant cultures. TH-immunohistochemistry over the tissue slice area demonstrates that the TH-positive neurons appeared healthy in control culture (a), and cultures from CD47^−/−^ (b), SIRPα mutant (c), and control culture treated with antibodies against TSP-1 (d). Measurements of TH-positive nerve fiber density (e), length (f), and astrocytic migration revealed similar values for SIRPα wildtypes and mutants. Scale bar = 25 µm.

### Dissection for Ventral Mesencephalic Culture Preparation

Pregnant CD47^−/−^, SIRPα mutants, and their wildtype (WT) mice were deeply anesthetized using isoflurane (Baxter Medical AB; Sweden). The ventral mesencephalic (VM) area of the fetal brain was dissected under a microscope in sterile conditions. For the dissection, Dulbecco’s modified Eagle’s medium (DMEM; Gibco, Invitrogen, Stockholm, Sweden) was used. The dissected tissue pieces were sliced into 300 µm coronal sections using a tissue chopper and transferred to DMEM. The tissue slices was cut in the midline into two pieces and for each culture, one such piece was used. These tissue slices were attached to sterile, poly-d-lysine coated (5 mg/100 ml dH_2_O; Sigma-Aldrich; Stockholm, Sweden) coverslips (12×24 mm) in two drops of chicken plasma (Sigma Aldrich) and one drop of thrombin (1,000 units/ml; Sigma-Aldrich). The tissue slice/plasma/thrombin mixture was dried for 15–20 min before placed in 15 ml Falcon tubes containing 0.9 ml of medium. The tubes were inserted in a “roller-drum” placed in an incubator at 37°C in 5% CO_2_. The cultures were continuously rotating at a speed of 0.5 turns per minute.

**Figure 4:**
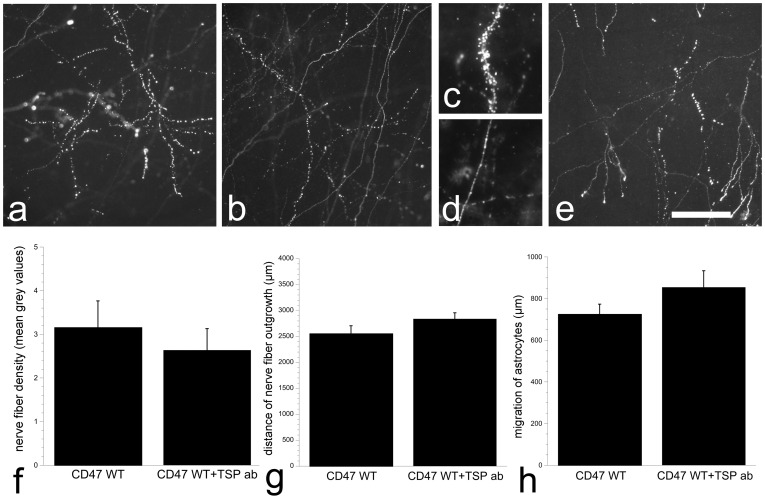
Effects of treatment with TSP-1 antibodies. Non-glial-associated nerve fiber outgrowth in control culture (a), cultures from SIRPα mutant (b, c, d), and control cultures treated with antibodies against TSP-1 (e) as revealed by TH-immunohistochemistry. In all cultures, dotted nerve fibers were present, and in higher magnification it is clear that the TH-immunoreactive dots have diffused from the axon and is also present in the near vicinity of the axon (c), while in non-disrupted axons TH-immunoreactivity stays within the nerve fiber (d). Neither nerve fiber density (f) nor the distance that nerve fibers reached (g) nor astrocytic migration (h) were affected by treatment with antibodies against TSP-1. Scale bar a, b, e = 70 µm, c, d = 25 µm.

The medium contained 55% DMEM (Gibco), 32.5% Hanks’ balanced salt solution (Gibco), 10% fetal bovine serum (Gibco), 1.5% glucose (Gibco), and 1% Hepes (Gibco). All ingredients were mixed and filtered through a sterile filter (pore size 0.22 µm; Sterivex, Millipore, Stockholm, Sweden). Antibiotics were added to the medium in a final concentration of 1% (10,000 units/ml penicillin, 10 mg/ml streptomycin, 25 µg/ml amphotericin; Gibco) and used at plating. At the first medium change and thereafter, antibiotics were excluded. The medium was changed twice a week.

The tissue slices were kept in culture for 7 (CD47^+/+^ n = 20; CD47^−/−^ n = 9) or 14 (CD47^+/+^ n = 33; CD47^−/−^ n = 14; SIRPα mutants n = 19; SIRPα WT n = 6) days. The n’s were based from several experiments: 7 DIV CD47^+/+^ n = 4; CD47^−/−^ n = 5 and 14 DIV: CD47^+/+^ n = 7; CD47^−/−^ n = 6; SIRPα mutants n = 4; SIRPα WT n = 2. Antibodies against thombospondin-1 (TSP-1; mAb-4; Clone A6.1; mouse IgG1; 2.5 µg/ml, n = 10, produced from 6 experiments) were added to the medium of cultures derived from CD47^+/+^ tissue from plating and throughout the experiment. Antibodies were purified from hybridoma supernatants by ammonium sulfate precipitation and affinity chromatography using Protein G High Trap columns (Amersham Bioscience, Piscataway, NJ). Control cultures to the antibody treatment were treated with vehicle added to the medium of CD47^+/+^ tissue.

### Immunohistochemistry

At 7 or 14 days *in vitro* (DIV), the ventral mesencephalic cultures were fixed in 2% paraformaldehyde in 0.1 M phosphate buffer (pH = 7.4) for 1 h and rinsed 3 times for 10 min in phosphate buffered saline (PBS; pH = 7.4) at room temperature. Afterwards, the cultures were incubated in antibodies raised against tyrosine hydroxylase (TH; diluted 1/300; rabbit anti-rat; Pel-Freez, Rogers, AR, USA), and in antibodies against vimentin to visualize astrocytes (diluted 1/200, mouse monoclonal, clone V9, Sigma Aldrich, Stockholm, Sweden; or diluted 1/200, chicken anti-rat, Abcam, Cambridge, UK) for 48–72 h at 4°C. After rinsing in PBS, cultures were incubated in secondary antibodies Alexa 594 (diluted 1/500; goat anti-rabbit; Molecular Probes Inc., Eugen, OR, USA) and Alexa 488 (diluted 1/500; goat anti-mouse or goat anti-chicken diluted 1/200; Molecular Probes) for 1 h at room temperature. For staining of cell nuclei, the cultures were incubated with DAPI (diluted 1/50, Molecular Probes) for 10 min at room temperature. All antibodies and DAPI were diluted in 1% Triton X-100 in PBS. Incubations were performed in a humidified atmosphere. All cultures were triple labeled, which was performed in sequence with one antibody at the time. Controls for unspecific immunohistochemistry were performed where the primary antibodies were omitted. After additional rinsing, the cultures were mounted in 90% glycerin in PBS.

### Image Analysis and Statistics

Image analysis was used to measure the distances that astrocytes had migrated and the length of TH-positive nerve fiber outgrowth. The distances were measured from the periphery of the tissue slice to the distal end that nerve fibers or the migrating astrocytes had reached using a microscale mounted in one of the oculars. Estimations of overall TH-positive outgrowth were based on 3–4 measurements in each culture over areas that displayed nerve fiber outgrowth, and estimations of vimentin-positive astrocytes were performed on measurements made in 4 perpendicular directions from the tissue slice. Nerve fiber density measurements were performed using NIH image analysis program in images captured using a 20X lens and a CCD camera (Jena Optic, Jena, Germany). The density measurements were performed over areas distal to the migration of astrocytes, near the astrocytic borderline. DAPI staining confirmed that no other cell types than vimentin-positive had migrated from the tissue slice. All measurements were performed on blind-coded slides. Images for publication were captured using a Retiga-4000RV CCD camera (Q-Imaging, Surrey, BC, Canada) and processed with Openlab software (Improvision, Cambridge, UK).

Statistical analysis were performed on means per slice culture and type of measurement using two-factor analysis of variance (ANOVA) for genotype and DIV interactions, and the analysis was followed by post hoc analysis using Student’s t-test. All results were expressed as means ± SEM, and significant level was set at p<0.05.

## Results

### CD47 Gene Deletion Affects TH-positive Nerve Fiber Outgrowth

VM tissue slices from E14 CD47^+/+^ and CD47^−/−^ fetuses were cultured for 7 and 14 DIV and examined for TH- and vimentin-immunohistochemistry. The results revealed that the distance for TH-positive nerve fiber outgrowth was affected by time without any significance in interaction between time and genotypes (time: F_1, 48_ = 47.34, p<0.00; time×genotype: F_1, 48_ = 0.56, p = 0.458; two-factor ANOVA). No difference in the length of outgrowth was found between CD47^+/+^ and CD47^−/−^ at 7 DIV, while at 2 weeks the TH-positive nerve fibers had reached significantly longer distances in cultures derived from CD47^−/−^ than in CD47^+/+^ cultures (Figs. 1, 2a, and Fig. S1; t_43_ = 3.234, p<0.01). The total distance reached by the nerve fiber outgrowth was around 1 mm at 7 DIV and around 3.5 mm at 14 DIV for CD47^−/−^ cultures, which was longer than the astrocytic migration had reached at the respective time points (Fig. 2c). The density of TH-positive nerve fibers was significantly higher in CD47^−/−^ compared to control cultures at 14 DIV (t_45_ = 4.673, p<0.001; Fig. 2b, d, e). Furthermore, in all cultures, the TH-positive neurons had a healthy appearance (Fig. 3a, b). However, in CD47^+/+^ cultures the TH-positive nerve fibers had a dotted appearance at 14 DIV, while thin nerve fibers with enlarged nerve endings were found in cultures derived from CD47^−/−^ (Fig. 2d-e, inserts). This dotted appearance were not present at 7 DIV.

The astrocytes, as determined by vimentin-immunoreactivity, were migrating from the tissue slice and formed a monolayer surrounding the slice. The distance that the astrocytes had migrated at 7 DIV was around 300 µm with no difference between CD47^+/+^ or CD47^−/−^ cultures. However, time had effect on the distances reached by migrating astrocytes (F_1, 48_ = 47.344, p<0.001; two-factor ANOVA) and within each genotype significantly enhanced length was reached at 14 DIV (CD47^+/+^: t_30_ = 6.743, p<0.001; CD47^−/−^: t_14_ = 3.604, p<3.604), while no difference was found between genotypes (Fig. 2c).

### SIRPα Mutation did not Affect TH-positive Nerve Fiber Outgrowth

To further investigate the influence by CD47 on nerve fiber growth, the CD47 receptor SIRPα was investigated, and cultures from SIRPα mutants were studied at 14 DIV. TH-positive neurons appeared healthy in both mutant and wildtype cultures, and nerve fiber outgrowth reached similar distances (p = 0.574; Fig. 3c, f). The TH-positive nerve fibers had similar morphology in both SIRPα mutant and their wildtype cultures as in the CD47^+/+^ cultures, i.e. some nerve fibers were thin and smooth while others were discontinuous and appeared as dotted lines, with TH-positive dots spread surrounding the nerve fibers (Fig. 4a–d). The density of TH-positive nerve fibers was not affected by SIRPα mutation (p = 0.360; Fig. 3e). In addition, migration of vimentin-positive astrocytes did not differ between genotypes (p = 0.418; Fig. 3g).

### Blocking the Effect of CD47 by Treatment with Antibodies Against TSP-1

The results from SIRPα mutant cultures suggested that the enhanced nerve fiber growth found in cultures derived from CD47 gene deleted tissue was not an effect mediated through a block of CD47-SIRPα interaction. Therefore antibodies against TSP-1 were added to CD47^+/+^ culture medium to study possible CD47-TSP-1 interactions. The results revealed that the TH-positive neurons appeared healthy (Fig. 3d). Neither TH-positive nerve fiber outgrowth (p = 0.442) nor nerve fiber density (p = 0.646) was affected by adding antibodies against TSP-1, and again there were elements of dotted TH-positive nerve fibers in most cultures (Fig. 4e–g). Migration of vimentin-positive astrocytes was not affected by the antibody treatment (p = 0.156; Fig. 4h).

## Discussion

The present study demonstrates that in the absence of CD47, nerve fiber growth is robust, may grow exceeding 3 mm of distance including growth both onto migrating astrocytes and beyond the distance that the astrocytes had reached in 2 weeks without any degenerative signs. These non-glial-associated nerve fibers are generally retracting at 14 DIV, as found also in the CD47^+/+^ cultures, when the astrocytes have migrated and formed a monolayer surrounding the tissue slice, suggesting that the astrocytes were permissive for these non-glial-associated nerve growth in the absence of CD47. Thus, it is possible to attenuate the retraction of the non-glial-associated growth, earlier found in both rat and mouse organotypic slice cultures, by blocking the expression of CD47.

The non-glial-associated nerve fibers are found in organotypic slice cultures when the tissue is attached to the substrate already at plating [5], [31], [32]. It has been demonstrated that the age of the tissue at plating influences the presence of the non-glial-associated growth such that the earlier the stage of the tissue at plating, the more robust is the expression [6]. Therefore, E14 was used in the present study as an optimal time point for when the non-glial-associated growth is first developed and then retracted [5]. The retraction might be interpreted as degeneration, however, this has not been possible to prove due to different time schedules for apoptotic markers at the cell body level and axon disruption, which occurs later [10]. From previous studies, it appears to be a tight correlation between the astrocytic migration and the presence of non-glial-associated growth, such that when the astrocytes migrate, the non-glial-associated growth disappears earlier and it is stimulated when the astrocytes are inhibited [6], [7], [8], [33]. Thus, the correlation between the non-glial- and glial-associated nerve fibers appears always to end up in the more persistent glial-associated growth, but over different time schedule depending on treatment. Although this study has not included the glial-associated growth, due to difficulties to distinguish between the two growth patterns, because the non-glial-associated nerve fibers were too robust in the CD47^−/−^ cultures, the presence of astrocytes was similar in all cultures. This is the first time that no sign of retraction of the non-glial-associated nerve fibers has been documented in the presence of astrocytic migration at 14 DIV, as found in the CD47^−/−^ tissue cultures. Interestingly, the lack of CD47 enhanced both the length and density of the non-glial-associated nerve fibers, parameters that has never been affected before.

To study possible mechanisms that promoted nerve fiber growth in the absence of CD47, cultures from the CD47 receptor SIRPα mutants were investigated. SIRPα is a transmembrane protein with an extracellular domain that binds to CD47 and a cytoplasmatic domain containing four tyrosine phosphorylation sites that serve as binding sites for the Src homology 2 domains of SHP-1 and SHP-2 [34]. The SIRPα mutant mouse has the normal extracellular domain, but truncated intracellular domain of the receptor and can thus not mediate the signaling following CD47 binding [30]. SIRPα is highly expressed in neurons and enriched in growth cones, may take part in the regulation of both neurite outgrowth and synapse formation via its binding to CD47 [34], [35], [36]. However, in the present study, no significant effect on nerve fiber growth was found when comparing SIRPα mutant to control cultures, which indicates that lack of CD47 binding to SIRPα, and subsequent lack of SIRPα signaling, could not explain the enhanced nerve growth in the CD47^−/−^ cultures. Thus, the hampered outgrowth found in the presence of CD47 is unlikely to be mediated by signaling through SIRPα. However, it is known that the extracellular domain of SIRPα can be cleaved and released in the cultures from SIRPα mutants [37], [38]. Furthermore, CD47 can bind to SIRPα, creating a bidirectional-signaling complex such that the signal may be mediated through CD47 [39], [40]. Thus, an interaction between the extracellular domain of SIRPα and CD47 in the SIRPα mutant cultures might still be active and could explain why no difference is seen between SIRPα mutant compared to their wildtype cultures.

To further study the pathways, by which CD47 may exert its action in the control cultures, functional blocking antibodies against TSP-1 were added to the medium, where TSP-1 act as a ligand for CD47 [41]. Blocking TSP-1 revealed no change in nerve fiber formation or astrocytic migration, as compared to control cultures. Thus, the limited growth of non-glial-associated nerve growth in the presence of CD47 in control cultures is neither mediated via signaling SIRPα nor via CD47 signaling through TSP-1 binding. Interestingly, both TSP-1 and SIRPα are interacting with CD47 during synaptogenesis [17], [26], [38], [42], which means that other mechanisms appear to be involved in CD47-dependent regulation of the type of nerve fiber growth investigated in the present study. Thus, CD47 might exert its action via yet unknown ligands or receptors, indirect via producing factors that make the astrocytes permissive for the non-glial-associated nerve fibers, or by mechanisms regulated by signaling from CD47 itself.

### Conclusion

The presence of CD47 may affect nerve growth in control cultures, since nerve fiber outgrowth is seen also in the controls, however, it is clear from the present study that the absence of CD47 significantly improves nerve fiber growth *in vitro*. Furthermore, the hampered outgrowth seen in control cultures in the presence of CD47 appears not to be mediated via SIRPα or TSP-1. As this non-glial-associated nerve fiber growth is withdrawn when astrocytes proliferate and migrate from the tissue slice in normal culture, CD47 seems to affect the astrocytes rather than the nerve fibers. This has also been demonstrated by inhibiting the astrocytic proliferation where the drawback of non-glial-associated growth is abolished as long as the astrocytic migration is hampered [6]. Thus, the absence of CD47 may prevent drawback of the axons that form the non-glial-associated nerve fibers.

## Supporting Information

Figure S1 **TH-positive nerve fiber growth and vimentin-positive astrocytic migration.** To clarify that there is no co-existence between TH- (a, c) and vimentin- (b, d) –immunoreactivity in the tissue slices at 7 DIV, these images are here shown separately to complement Fig. 1. Comparing TH-positive nerve fiber outgrowth (a, c) with vimentin-positive astrocytic migration (b, d) demonstrates that the nerve fibers have reached longer distances than the migrating astrocytes. There is no difference between CD47^+/+^ (a, b) and CD47^−/−^ (c, d) cultures. Scale bar = 100 µm.
(TIF)
