# Supplementary figures and images for: The Absence of CD47 Promotes Nerve Fiber Growth from Cultured Ventral Mesencephalic Dopamine Neurons
Source: PLoS One. 2012 Sep 26;7(9):e45218. doi: 10.1371/journal.pone.0045218 (PMC3458886; doi:10.1371/journal.pone.0045218)

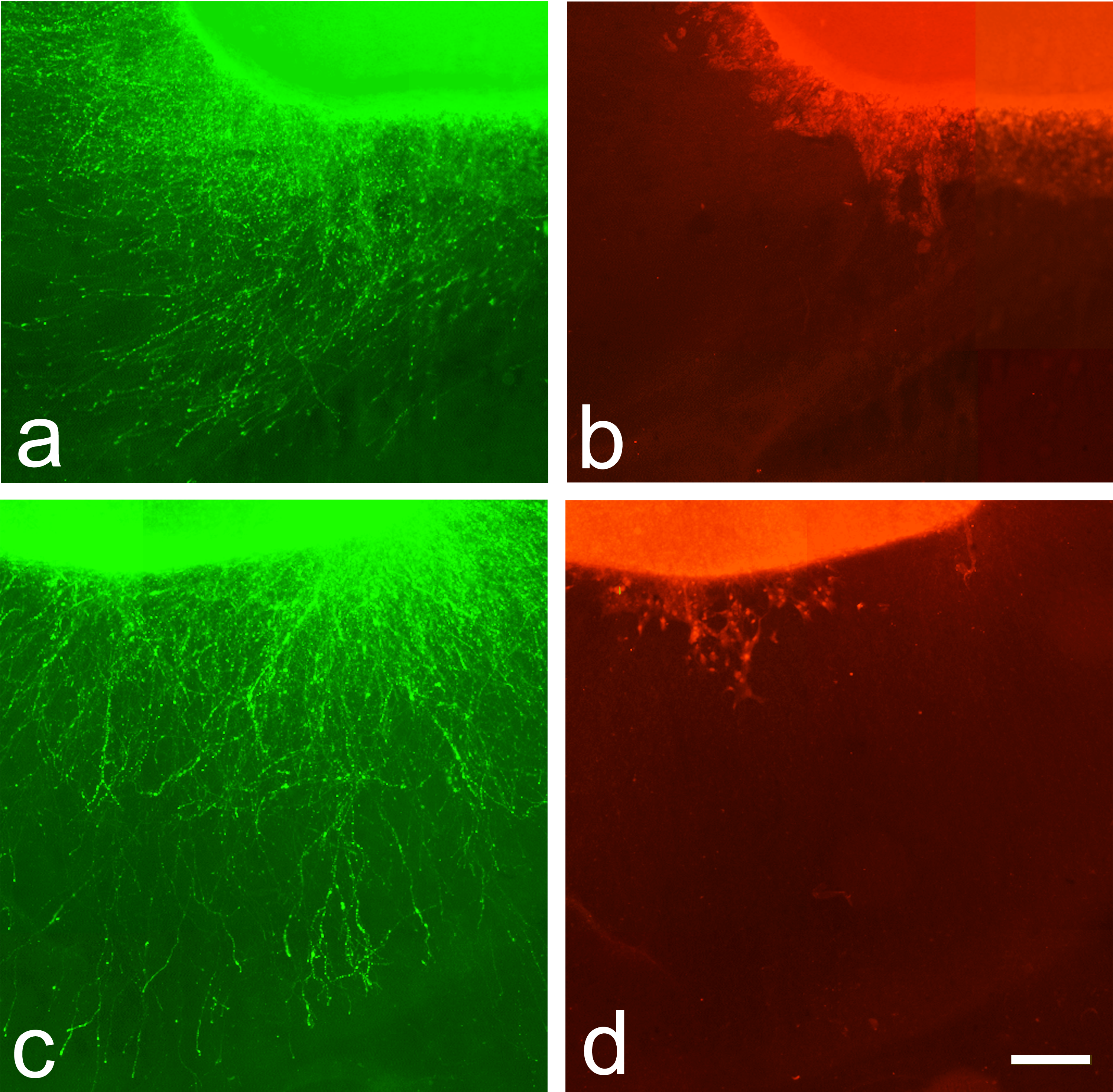

Supplement: Figure S1 — TH-positive nerve fiber growth and vimentin-positive astrocytic migration. To clarify that there is no co-existence between TH- (a, c) and vimentin- (b, d) –immunoreactivity in the tissue slices at 7 DIV, these images are here shown separately to complement Fig. 1. Comparing TH-positive nerve fiber outgrowth (a, c) with vimentin-positive astrocytic migration (b, d) demonstrates that the nerve fibers have reached longer distances than the migrating astrocytes. There is no difference between CD47+/+ (a, b) and CD47−/− (c, d) cultures. Scale bar = 100 µm. (TIF) [file pone.0045218.s001.tif]
